# Impact of Graft Tunnel Placement on Short-Term Clinical Outcome Following Anterior Cruciate Ligament Reconstruction

**DOI:** 10.5704/MOJ.2507.006

**Published:** 2025-07

**Authors:** M Rogger, O Al-Dadah

**Affiliations:** 1 Department of Trauma and Orthopaedics, South Tyneside District Hospital, South Shields, United Kingdom; 2 Translational and Clinical Research Institute, Faculty of Medical Sciences, Newcastle University, Newcastle-upon-Tyne, United Kingdom

**Keywords:** anterior cruciate ligament, patient-reported outcome measures, graft tunnel placement, reconstruction, radiograph

## Abstract

**Introduction:**

Anterior cruciate ligament (ACL) tears are a common musculoskeletal injury often requiring anterior cruciate ligament reconstruction (ACLR). Many factors are thought to influence patient outcome and determining the extent can allow for optimisation of patient care. One of these factors is graft tunnel placement, both femoral and tibial. The aim of this study was to investigate whether graft tunnel placement influences clinical outcome following ACLR.

**Materials and Methods:**

The patient responses from six patient-reported outcome measures (PROM) at initial presentation and one year following ACLR, as well as demographic data at presentation, were collected. Graft tunnel placement was evaluated using 10 validated radiological measurements on antero-posterior and lateral radiographs following surgery.

**Results:**

A total of 45 patients were included in the study. There was a significant longitudinal improvement (p<0.001) for almost all PROM scores when comparing pre-operative to post-operative results. Overall, no significant correlation was demonstrated between graft tunnel placement and PROM scores, except for a weak association between femoral tunnel positioning on lateral view radiographs and the overall Knee injury and Osteoarthritis Outcome Score (rho=0.37, p=0.038) and the Lysholm score (rho=0.36, p=0.034) and also tibial tunnel placement on lateral view radiographs and the EQ-5D VAS score (rho=0.37, p=0.037).

**Conclusion:**

ACLR is a clinically successful treatment strategy for patients with symptomatic ACL tears. Graft tunnel positioning does not generally affect clinical outcomes, although there may be a weak association with femoral tunnel positioning on lateral radiographs.

## Introduction

Anterior cruciate ligament (ACL) tears often require surgical intervention to improve stability, restore function of the knee joint and reduce the risk of further injuries^1^. This is the preferred management option for individuals with symptomatic tears that are refractory to conservative treatment^2^. Non-surgical approaches may be adopted for less symptomatic tears with limited impact on activities of daily living or recreational activities, which primarily consists of bracing and physiotherapy rehabilitation^2^.

The most common intervention for ACL tears is anterior cruciate ligament reconstruction (ACLR)^3^. ACLR most commonly utilises autograft to reconstruct the ACL by securing it via tunnels into the patient’s femur and tibia^1^. The most frequently used grafts are hamstring or bone-patellar tendon-bone (BPTB) autografts^1^. Allografts and quadriceps tendon autografts are viable alternatives, although they are not commonly used for primary ACLR in the UK^1-3^.

An important factor of ACL reconstruction is graft tunnel placement within the femur and tibia. Tunnel positioning can be controlled during surgery and could be standardised if a certain position were to yield better patient outcomes. Research into the impacts of tunnel placement has not led to clear conclusions.

Some studies suggest that more posterior femoral tunnel positioning leads to better functional outcomes^4-5^. Similarly, Behrend *et al*^6^ found that femoral tunnels positioned too anteriorly along the femoral intercondylar line in lateral view radiographs could lead to worse patient outcomes. Debnath *et al*^7^ investigated a range of radiographic measurements for assessing tunnel positioning, finding only femoral tunnel placement in lateral view radiographs to be significantly correlated with patient outcome, with worse outcomes if the tunnel was located too posteriorly along Blumensaat’s line. More anterior placement of the tibial tunnel on lateral radiograph was shown, by Padua *et al*^8^, to improve clinical outcome, specifically when using the IKDC and Lysholm scores where the impacts were statistically significant. This was replicated by Avadhani *et al*^9^ who found that tibial tunnels positioned between 35-46% from the anterior aspect of the tibial plateau resulted in better outcomes for patients with BPTB grafts, whilst too anteriorly placed tibial tunnels had worse outcomes. In contrast, Behrend *et al*^6^ found that tibial tunnel positioning had no statistically significant impact on patient outcomes when using BPTB grafts.

The aim of this study was to investigate whether graft tunnel placement influences clinical outcome following ACLR. The hypothesis of this study is that more anterior placement of either the tibial or the femoral tunnel would result in poorer clinical outcomes.

## Materials and Methods

All the patients included in this longitudinal observational study attended a specialist knee clinic and subsequently underwent arthroscopic ACLR surgery by a single consultant orthopaedic surgeon with a specialist interest in knee surgery between October 2015 and March 2019. This study was exempt from institutional review board / ethics committee approval as it was a pragmatic study evaluating the existing clinical practice of the senior author. This observational research study constituted part of the first author’s Masters dissertation. Sample size calculation was not performed as all patients were included who fulfilled the inclusion criteria within the timeframe of the study.

Inclusion criteria comprised patients aged 16 years and over with a symptomatic ACL tear of the knee that was refractory to initial conservative treatment (i.e. physiotherapy) and subsequently underwent ACLR between October 2015 and March 2019. All patients had either a hamstring or bone-patellar tendon-bone (BPTB) autograft used for their ACLR followed by a standardised post-operative physiotherapy ACL rehabilitation pathway for all patients including immediate full weight bearing and full range of movement, unless the procedure was accompanied by a meniscus repair in which case a knee brace restricting range of movement from 0° to 90° for 6 weeks was used to protect the meniscus repair. Return to contact sports was only permitted 12 months after surgery. Patients with concomitant medial collateral ligament (not requiring any direct surgical intervention), meniscal or focal cartilaginous injuries were not excluded from the study. Exclusion criteria comprised patients with concurrent posterior cruciate ligament, lateral collateral ligament / postero-lateral corner ligament complex tears, revision ACLR surgery, Grade III or IV degenerative changes according to Outerbridge Classification^10^ and patients who did not attend their post-operative clinic appointment and complete their one-year follow-up patient reported outcome measure (PROM) questionnaires.

All patients underwent arthroscopic ACLR using the anatomic single-bundle technique. For patients undergoing ACLR using BPTB grafts, the trans-portal technique was used to create the femoral tunnel at the centre of the femoral footprint. The tibial tunnel was drilled using a tibial jig at 60°, which was aimed towards the centre of the tibial footprint of the ACL. SoftSilk interference screws [Smith and Nephew Inc., Andover, Massachusetts, USA] were used to secure the graft both in the femur and the tibia. The graft was then tensioned with the knee in 20° flexion.

For the hamstring graft approach, the trans-portal technique was used to drill the femoral tunnel at the centre of the femoral footprint. The tibial tunnel was created using the tibial jig at 55° aimed at the centre of the tibial footprint of the ACL. Hamstring grafts were secured to the femur via suspensory fixation using EndoButton [Smith and Nephew Inc., Andover, Massachusetts, USA] and to the tibia using radiolucent polyetheretherketone (PEEK) interference screws [Smith and Nephew Inc., Andover, Massachusetts, USA] or round cannulated interference (RCI) screws [Smith and Nephew Inc., Andover, Massachusetts, USA]. This is the surgeon’s preferred technique when operating on patients with cosmetic preferences and patients who are required to be able to kneel for extended periods during occupational, religious or recreational activities, as there is a lower theoretical risk of anterior knee pain as compared to the BPTB technique although the latter graft is recognised as being a biomechanically stronger graft.

Demographic details and information relating to the injury were obtained for all the patients through medical records, PROM questionnaires and operative notes. The preoperative PROM questionnaire was completed at the initial outpatient clinic consultation with the surgeon. The postoperative PROM questionnaire was completed at the patient’s one year follow-up clinic consultation with the surgeon.

The following validated PROMs were included: Knee injury and Osteoarthritis Outcome Score (KOOS)^11^, International Knee Documentation Committee (IKDC) subjective knee score^12,13^, Lysholm Score^14^, Tegner Activity Score^14^, the 12-Item Short Form Health Survey (SF-12), divided into physical component summary (PCS) and mental component summary (MCS)^15^ and EuroQol-5D (EQ-5D-5L), including both the index score and visual analogue scale (VAS)^16-18^.

**Fig. 1:**
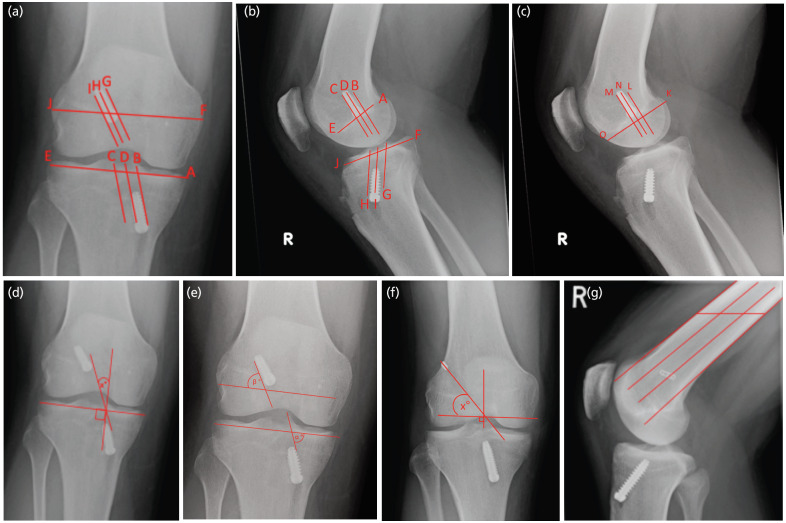
Graft tunnel placement measurements. (a) Femoral and tibial tunnel placements calculations in AP view. (b) Femoral tunnel placement along Blumensaat’s line (AE) and tibial tunnel placement along tibial plateau. (c) Femoral tunnel placement along femoral intercondylar line. (d) Graft inclination angle calculation method. (e) Alpha (_a_) and Beta (b) angles. (f) Clockface method. (g) EndoButton placement, this image shows middle placement of the EndoButton.

Radiographic information for analysis was obtained through the Picture Archiving and Communication System (PACS) [Centricity version 6; GE Healthcare, Chicago, IL, USA]. Both antero-posterior (AP) and lateral radiographs following ACLR surgery were evaluated and included in the analysis. All measurements were undertaken by the first author according to the following validated methods. Fig. 1 illustrates the measurement techniques.

Tunnel positions in the AP view, as seen in Fig. 1a, were previously used in a range of studies, and are validated^4,7,9^. Femoral tunnel placement, AP view (Fig. 1a). To obtain the position of the femoral tunnel in AP view, the distance between the widest points of the femoral tunnel must be obtained (JF). The slightly sclerotic margins of the tunnel (G and I) can then be marked to find the midpoint of the tunnel (H). Positioning is expressed as a percentage of FJ to allow for standardisation amongst patients. This is calculated as (JH/JF)x100.

Tibial tunnel placement, AP view (Fig. 1a). Initially, a straight line across the tibial plateau is drawn (AE). The sclerotic margins of the tibial tunnel are marked (B and C). The midpoint of the tibial tunnel is obtained (D). The positioning of the tibial tunnel is expressed as a percentage across the tibial plateau, (AD/AE)x100.

When measuring femoral tunnel placement along Blumensaat’s line in the lateral view (Fig. 1b), draw a line along Blumensaat’s line (AE), a sclerotic line located in the femoral condyle. Find the midpoint of the femoral tunnel (D). The equation (AD/AE)x100 is used to obtain tunnel position as a percentage along Blumensaat’s line. This is a commonly used and validated method for measuring femoral tunnel placement^4-8^.

When calculating the tibial tunnel placement in a lateral view (Fig. 1b), a horizontal line parallel to the length of the tibial plateau is drawn (FJ). The sclerotic margins of the tibial tunnel are identified and marked (G and H). They are then used to obtain the midpoint of the tibial tunnel (I). Using the equation (JI/JF)x100, the tibial tunnel position is found as a percentage in relation to the tibial plateau. This is a validated method, which has been used frequently in the literature^4-8^.

Another measurement used for femoral tunnel placement was along the femoral intercondylar line. A line along Blumensaat’s line (KO) is drawn across the whole length of roof of the intercondylar notch. The sclerotic margins of the femoral tunnel (L and M) are used to obtain the tunnel midpoint (N). Using the equation (KN/KO)x100, femoral tunnel positioning is expressed as a percentage across the total intercondylar distance, which was previously described by Padua *et al*^8^ (Fig. 1c).

Graft inclination was calculated to establish the potential influence of the combined orientations, which was a method previously used by Debnath *et al*^7^. To obtain the graft inclination angle, as seen in Fig. 1d, a line connecting the medial aspects of the femoral and tibial tunnel is drawn. The angle between this line and a vertical line perpendicular to the tibial plateau is the graft inclination (x°) (Fig. 1d).

Angulation of the individual tunnels were measured (Fig. 1e). The alpha (a) angle relates to the tibial tunnel and has been validated by Behrend *et al*^6^ and Padua *et al*^8^. To obtain the alpha angle, a horizontal line across the tibial plateau must be drawn. Then, a straight line along the medial border of the tibial tunnel is drawn. The angle between this line and the tibial plateau will give the alpha (a) angle, as seen in Fig. 1e. Padua *et al*^8^ also described the beta (b) method, as illustrated in Fig. 1e. The beta angle is calculated by drawing a horizontal line across the femoral condyles parallel to the tibial plateau. Then a straight line along the lateral aspect of the femoral tunnel is drawn. The angle between the two lines (β) is obtained, as illustrated in Fig. 1e.

The clockface method was described by Behrend *et al*^6^ and Kazemi *et al*^19^, to quantify how steeply the femoral tunnel has been drilled. The approach was modified in this study as a ‘clockface’ was not available for insertion in the PACS software. The modified measurements are depicted in Fig. 1f. A horizontal line across the tibial plateau is drawn with a perpendicular line through it. This cross is then placed in the centre of the femoral intercondylar notch. A line is drawn from the centre of the cross to the centre of the EndoButton or screw. The angle between the horizontal line and the bisecting line is used to calculate angle x°. The values are divided into two groups – low and high positioned groups. Low positioned being between 30° and 45° and the high positioned group had values between 45° and 60°. This was in line with the methods described by Kazemi *et al*^19^.

EndoButton placement was only measured for patients who underwent ACLR using a hamstring tendon autograft (Fig. 1g). As described by Gunaydin *et al*^20^, EndoButton placement on lateral radiographs were categorised into three discrete sections and compared in this way. For this measurement, the femur must be divided into three equal parts to enable the classification of the EndoButton location. Two straight lines along the cortices of the femur are drawn. To divide the femur into three equal sections, a line parallel to the ground is drawn and the length of this line is found. The length is divided by three and lines through the femoral shaft are drawn. The EndoButton is then assigned either anterior, middle, or posterior location based on where the majority of the EndoButton is located as seen in Fig. 1g.

Plotted histograms with fitted curve lines, boxplots, normal Q-Q plots, and the Shapiro-Wilk statistic were used to test the data for normal distribution. All the radiological measurement data (continuous variables) showed a normal distribution. However, almost all the PROM data (continuous variables) displayed a skewed distribution and therefore the relevant non-parametric statistical tests were used for the analysis. The level of statistical significance was set at p<0.05. Two-tailed asymptotic p-values were used for the analysis. Statistical analysis was performed using IBM SPSS Statistics for Windows version 26.0 [IBM Corp., Armonk, New York].

## Results

Table I shows the patient demographics of the study cohort. There were proportionally more males than females in the study.

**Table I:** Demographics of patients.

	Patients (n=45)
Age, years [mean (SD)]	32.4 (11.9)
Gender (Female : Male)	11 : 34
Laterality (Right : Left)	21 : 24
BMI [mean (SD)]	27 (5.1)
Height (m) [mean (SD)]	1.76 (0.09)
Weight (kg) [mean (SD)]	84.2 (16.2)
Smoker (Yes : No)	5 : 40
Graft type (hamstring : BPTB)	30 : 15
ACL tear (full : partial)	38 : 7
MCL tear (Yes : No)	10 : 23
Meniscal tear (Yes : No)	32 : 13
Cartilage Lesion (Yes : No)	8 : 25
Tegner activity score prior to injury [median(IQR)]	7.0 (6.0 - 9.0)

Abbreviations - SD: standard deviation, IQR: inter-quartile range, BMI: body mass index, BPTB: bone-patellar tendon-bone, MCL: medial collateral ligament

The clinical outcome analysis (Table II) demonstrated a statistically significant improvement following ACLR surgery for all PROM scores, except for SF-12 MCS (p=0.070).

**Table II:** Longitudinal PROM scores analysis before and after ACLR.

	Pre-op [median(IQR)] n=45	Post-op [median(IQR)] n=45	p-value^1^	Z
KOOS				
Pain	58.3 (50-73.6)	91.7 (80.6-97.2)	<0.001*	-5.065
Symptoms	57.1 (42.9-7.4)	82.1 (71.4-89.3)	<0.001*	-4.735
ADL	69.1 (55.9-80.9)	97.8 (87.1-100.0)	<0.001*	-5.148
Sport/Rec	30.0 (15.0-50.0)	80.0 (60.0-87.5)	<0.001*	-4.607
QoL	18.8 (12.5-29.7)	68.8 (56.3-75.0)	<0.001*	-5.452
Overall	48.7 (37.4-61.2)	82.5 (72.2-87.8)	<0.001*	-5.016
IKDC	41.4 (29.9-50.3)	75.9 (67.8-88.5)	<0.001*	-5.471
Lysholm	53.0 (37.0-65.8)	88.5 (79.0-93.0)	<0.001*	-5.604
Tegner	2.0 (1.3-3.0)	6.0 (4.0-7.0)	<0.001*	-5.124
SF-12 PCS	35.0 (29.9-42.4)	53.4 (45.8-56.6)	<0.001*	-5.182
SF-12 MCS	52.9 (38.7-58.0)	55.0 (47.1-59.0)	0.070	-1.809
EQ-5D Index	0.596 (0.450-0.732)	0.837 (0.736-1.00)	<0.001*	-4.785
EQ-5D VAS	70.0 (50.0-87.3)	88.0 (73.8-90.0)	<0.001*	-4.159

Notes - 1Wilcoxon Signed Ranks Test, *Statistically significant (p<0.05), pre-op: pre-operative, post-op: post-operative, IQR: interquartile range, KOOS: Knee injury and Osteoarthritis Outcome Score, ADL: activities of daily living, Sport/Rec: Sport and Recreation Function, QoL: quality of life, IKDC: International Knee Documentation Score, SF-12: Short Form 12, MCS: mental component summary, PCS: physical component summary, EQ-5D: EuroQol-5D, VAS: visual analogue score, PROM: patient reported outcome measure, ACLR: anterior cruciate ligament reconstruction

Table III shows the radiological measurements of continuous data variables, which include graft angles and inclination as well as the placements of both the tibial and the femoral graft tunnels.

**Table III:** Radiological measurements (continuous variables).

	n=36 mean (±SD)
Alpha angle (°)	74.79 (±9.17)
Beta angle (°)	46.30 (±10.51)
Graft inclination angle (°)	22.71 (±5.02)
Tibial tunnel placement (lateral view) (%)	41.33 (±5.95)
Tibial tunnel placement (AP view) (%)	43.49 (±3.23)
Femoral tunnel placement (AP view) (%)	32.41 (±5.18)
Femoral tunnel placement along Blumensaat’s line (lateral view) (%)	43.83 (±11.05)
Femoral tunnel placement along intercondylar line (lateral view) (%)	45.01 (±8.47)

Abbreviations - SD: standard deviation, AP: antero-posterior

Table IV, V and VI show the radiological measurements of discrete data variables. This includes the clockface method, categorisation based on EndoButton location and the combination of the two measurements.

**Table IV:** Femoral tunnel positioning using the clockface method (antero-posterior radiograph).

	Femoral tunnel, clockface method (n=36)	
	Low position (30-45°)	High position (45 -60°)
Frequency count	22	14

**Table V:** EndoButton placement (lateral radiograph).

	Endobutton placement (n=23)		
	Anterior	Middle	Posterior
Frequency count	9	13	1

**Table VI:** Combined EndoButton placement (lateral radiograph) with femoral tunnel placement using the clockface method (antero-posterior radiograph).

	Combined EndoButton and clockface method (n=23)					
	Low anterior	Low middle	Low posterior	High anterior	High middle	High posterior
Frequency count	6	10	0	3	3	1

Table VII shows the comparison of post-operative patient outcomes based on femoral tunnel placement using the clockface method (Fig. 1f). No statistically significant differences were found for whether the tunnel was considered low (0° – 45°) or high (45° – 60°).

**Table VII#:** Comparison of post-operative PROM scores comparing low versus high femoral tunnel placement using the clockface method (antero-posterior radiograph).

	Low [median (IQR)] n=22	High [median (IQR)] n=14	p-value^1^	Z	U
KOOS					
Pain	91.7 (79.9-94.4)	88.9 (75.0-95.8)	0.669	-0.427	118.5
Symptoms	82.1 (70.5-89.3)	83.9 (71.4-89.3)	0.719	-0.359	143.0
ADL	97.1 (84.6-99.3)	94.1(75.0-100.0)	0.798	-0.256	139.5
Sport/Rec	75.0 (65.0-85.0)	80.0 (50.0-87.5)	0.938	-0.077	121.5
QoL	68.8 (62.5-91.9)	68.8 (26.3-84.4)	0.256	-1.137	99.5
Overall	82.5 (69.4-84.6)	82.5 (69.9-89.8)	0.833	-0.211	118.0
IKDC	74.7 (67.8-86.2)	74.7 (62.6-85.6)	0.672	-0.423	112.5
Lysholm	88.5 (82.5-91.5)	83.0 (76.5-94.0)	0.707	-0.376	132.0
Tegner	6.0 (4.0-7.5)	5.0 (4.0-7.0)	0.301	-1.034	91.5
SF-12 PCS	51.7 (45.9-55.8)	50.2 (41.1-56.3)	0.749	-0.320	137.5
SF-12 MCS	52.5 (42.4-58.8)	57.6 (51.6-60.2)	0.102	-1.634	98.5
EQ-5D Index	0.837 (0.735-1.00)	0.837 (0.679-1.00)	0.679	-0.413	128.5
EQ-5D VAS	82.5 (70.0-90.0)	90.0 (75.0-90.5)	0.423	-0.801	108.5

Notes - ^1^Mann-Whitney U Test, #No statistically significant results, IQR: inter-quartile range, KOOS: Knee injury and Osteoarthritis Outcome Score, ADL: activities of daily living, Sport/Rec: Sport and Recreation Function, QoL: quality of life, IKDC: International Knee Documentation Score, SF-12: Short Form 12, MCS: mental component summary, PCS: physical component summary, EQ-5D: EuroQol-5D, VAS: visual analogue score

Table VIII shows the comparison of post-operative scores based on the location of the EndoButton in lateral view radiographs for patients who underwent an ACLR using hamstring tendon autograft (Fig. 1g). The only statistically significant difference was found to be the EQ-5D Index (p=0.029), where anterior positioning was found to have the best outcomes followed by middle placement. The number of patients in the posterior group was too small to compute an inter-quartile range (IQR).

**Table VIII:** Comparison of post-operative PROM scores based on EndoButton placement (lateral radiograph).

	Anterior [median (IQR)] n=9	Middle [median (IQR)] n=13	Posterior [median] n=1	p-value^1^	H
KOOS					
Pain	94.4 (55.6-100.0)	84.7 (59.0-93.8)	91.7	0.336	2.18
Symptoms	85.7 (62.5-89.3)	75.0 (53.6-87.5)	89.3	0.364	2.02
ADL	97.8 (78.3-100.0)	86.8 (64.7-98.5)	92.4	0.548	1.20
Sport/Rec	85.0 (60.0-95.0)	70.0 (45.0-75.0)	70.0	0.375	1.96
QoL	62.5 (25.0-93.8)	56.3 (37.5-67.2)	68.8	0.377	1.95
Overall	84.6 (56.6-94.6)	75.3 (43.6-81.3)	82.5	0.190	3.32
IKDC	74.7 (56.6-92.0)	71.3 (56.6-74.7)	67.8	0.525	1.29
Lysholm	90.0 (81.5-95.5)	79.0 (73.5-87.0)	92.0	0.093	4.74
Tegner	7.0 (5.0-8.0)	4.5 (2.8-7.5)	4.0	0.488	1.44
SF-12 PCS	56.1 (48.8-56.7)	44.7 (34.8-52.2)	43.6	0.074	5.21
SF-12 MCS	52.5 (41.1-59.8)	53.0 (44.9-59.5)	56.3	0.971	0.06
EQ-5D Index	1.00 (0.919-1.00)	0.745 (0.546-0.837)	0.679	0.029*	7.11
EQ-5D VAS	75.0 (60.0-85.0)	83.0 (70.0-90.0)	70.0	0.423	1.72

Notes - ^1^Kruskal-Wallis H Test, *Statistically significant (p<0.05), IQR: inter-quartile range, KOOS: Knee injury and Osteoarthritis Outcome Score, ADL: activities of daily living, Sport/Rec: Sport and Recreation Function, QoL: quality of life, IKDC: International Knee Documentation Score, SF-12: Short Form 12, MCS: mental component summary, PCS: physical component summary, EQ-5D: EuroQol-5D, VAS: visual analogue score

Table IX compares clinical outcomes following ACLR relating to femoral tunnel placement, with patients categorised based on a combination of EndoButton placement in lateral view radiographs (Fig. 1g) and the clockface method in AP view (Fig. 1f). There is no statistically significant difference between any of the locations of the femoral tunnel based on both views and PROM scores amongst the hamstring tendon autograft patients. The number of patients in some groups was too small to generate an IQR.

**Table IX#:** Comparison of post-operative PROM scores relating to tunnel placement based on a combination of EndoButton placement (lateral radiograph) and the femoral tunnel placement using clockface method (antero-posterior radiograph).

	Low anterior [median (IQR)] n=6	Low middle [median (IQR)] n=10	High anterior [median] n=3	High middle [median] n=3	High posterior [median] n=1	p-value^1^	Z
KOOS							
Pain	94.4 (69.4-100.0)	86.1 (65.3-94.4)	77.8	77.8	91.7	0.443	3.735
Symptoms	83.9 (65.2-90.2)	78.6 (55.4-86.6)	85.7	75.0	89.3	0.650	2.469
ADL	100.0 (92.6-100.0)	88.2 (73.9-98.5)	75.0	76.5	92.4	0.289	4.980
Sports	85.0 (70.0-92.5)	70.0 (53.8-75.0)	65.0	45.0	70.0	0.594	2.788
QoL	62.5 (43.8-87.5)	56.3 (43.8-65.6)	59.4	56.3	68.8	0.689	2.256
Overall	84.6 (69.6-91.7)	78.1 (50.0-81.2)	69.2	68.6	82.5	0.440	3.756
IKDC	74.7 (657-85.6)	71.8(59.4-75.3)	67.2	57.5	67.8	0.657	2.431
Lysholm	90.0 (85.5-93.3)	81.0 (73.8-86.5)	79.0	79.0	92.0	0.195	6.058
Tegner	7.0 (5.5-8.0)	4.0 (3.0-9.0)	4.0	5.0	4.0	0.641	2.520
SF-12 PCS	55.5 (50.1-56.9)	45.7 (37.0-51.2)	56.2	36.7	43.6	0.204	5.933
SF-12 MCS	51.5 (44.6-59.1)	47.7 (42.0-57.6)	59.8	60.0	56.3	0.530	3.171
EQ-5D Index	1.00 (0.959-1.00)	0.740 (0.576-0.919)	1.00	0.795	0.679	0.094	7.926
EQ-5D VAS	75.0 (62.5-85.0)	90.0 (70.0-92.5)	75.0	80.0	70.0	0.601	2.747

Notes - ^1^Kruskal-Wallis H Test, #No statistically significant values, IQR: inter-quartile range, KOOS: Knee injury and Osteoarthritis Outcome Score, ADL: activities of daily living, Sport/Rec: Sport and Recreation Function, QoL: quality of life, IKDC: International Knee Documentation Score, SF-12: Short Form 12, MCS: mental component summary, PCS: physical component summary, EQ-5D: EuroQol-5D, VAS: visual analogue score

Table X displays the correlation analysis between the radiographic measurements of graft tunnel placements described in Fig. 1a to 1c and the post-operative PROM scores following ACLR. Correlations between these measurements and PROM scores were obtained to identify whether there were any significant associations between tunnel placement and PROMs. There are no statistically significant correlations for any of the measurements in Table X except for femoral tunnel positioning along the intercondylar line in lateral view radiographs and tibial tunnel placement in the lateral view radiographs. For the femoral graft tunnel measurement, there are weak associations with the KOOS Sport/Rec sub-score (rho=0.37, p=0.037), KOOS Overall (rho=0.37, p=0.038) and Lysholm score (rho=0.36, p=0.034). Tibial tunnel placement in lateral view radiographs was largely not significantly associated with PROM scores, except for the EQ-5D VAS score (rho=0.37, p=0.037).

**Table X:** Correlation analysis between radiological measurements of graft tunnel positioning and post-operative PROM scores.

	Tibial tunnel (lateral view) rho p-value^1^ n=36	Tibial tunnel (AP view) rho p-value^1^ n=36	Femoral tunnel (AP view) rho p-value^1^ n=36	Femoral tunnel Blumensaat’s line (lateral view) rho p-value^1^ n=36	Femoral tunnel intercondylar line (lateral view) rho p-value^1^ n=36
KOOS					
Pain	0.23 (0.189)	-0.07 (0.711)	0.16 (0.376)	0.08 (0.642)	0.24 (0.184)
Symptoms	0.08 (0.644)	-0.01 (0.969)	0.06 (0.711)	0.14 (0.433)	0.27 (0.108)
ADL	0.08 (0.647)	0.08 (0.646)	0.19 (0.263)	0.05 (0.763)	0.21 (0.227)
Sport/Rec	0.06 (0.766)	-0.01 (0.980)	0.21 (0.256)	0.22 (0.236)	0.37 (0.037*)
QoL	0.11 (0.560)	0.07 (0.716)	0.24 (0.180)	0.04 (0.846)	0.21 (0.251)
Overall	0.09 (0.622)	0.12 (0.507)	0.25 (0.163)	0.18 (0.318)	0.37 (0.038*)
IKDC	0.17 (0.354)	0.21 (0.257)	0.12 (0.532)	0.22 (0.226)	0.34 (0.055)
Lysholm	0.12 (0.486)	0.10 (0.582)	-0.10 (0.956)	0.27 (0.117)	0.36 (0.034*)
Tegner	-0.24 (0.202)	-0.03 (0.863)	0.17 (0.354)	0.10 (0.592)	0.16 (0.398)
SF-12 PCS	0.15 (0.377)	0.21 (0.228)	0.14 (0.430)	0.04 (0.833)	0.18 (0.308)
SF-12 MCS	0.01 (0.949)	0.20 (0.253)	-0.03 (0.735)	0.10 (0.589)	0.13 (0.467)
EQ-5D Index	0.15 (0.404)	-0.05 (0.770)	0.10 (0.572)	-0.08 (0.661)	0.20 (0.260)
EQ-5D VAS	0.37 (0.037*)	-0.05 (0.796)	0.10 (0.598)	-0.16 (0.390)	-0.25 (0.161)

Notes - ^1^Spearman’s Rank Correlation Analysis, rho: Correlation Coefficient, *Statistically significant (p<0.05), AP: antero-posterior, KOOS: Knee injury and Osteoarthritis Outcome Score, ADL: activities of daily living, Sport/Rec: Sport and Recreation Function, QoL: quality of life, IKDC: International Knee Documentation Score, SF-12: Short Form 12, MCS: mental component summary, PCS: physical component summary, EQ-5D: EuroQol-5D, VAS: visual analogue score

Table XI shows the results of correlation analysis between the measurements of graft tunnel angles (Fig. 1d and 1f) and post-operative PROM scores. No statistically significant correlations were found in this analysis.

**Table XI#:** Correlation analysis between radiological measurements of graft tunnel angles and post-operative PROM scores.

	Alpha angle rho (p-value^1^) n=36	Beta angle rho (p-value^1^) n=36	Graft inclination rho (p-value^1^) n=36
KOOS			
Pain	-0.07 (0.701)	-0.04 (0.841)	0.16 (0.390)
Symptoms	-0.08 (0.662)	-0.02 (0.916)	0.06 (0.738)
ADL	-0.10 (0.856)	0.16 (0.354)	0.11 (0.517)
Sports	-0.10 (0.592)	0.02 (0.904)	0.12 (0.510)
QoL	-0.05 (0.805)	-0.04 (0.807)	-0.08 (0.653)
Overall	0.00 (0.997)	0.04 (0.817)	0.03 (0.878)
IKDC	-0.07 (0.723)	0.12 (0.526)	0.16 (0.389)
Lysholm	-0.10 (0.579)	0.10 (0.554)	0.26 (0.125)
Tegner	-0.08 (0.653)	-0.04 (0.838)	-0.09 (0.614)
SF-12 PCS	0.14 (0.435)	0.14 (0.437)	-0.02 (0.891)
SF-12 MCS	-0.19 (0.288)	-0.06 (0.735)	0.04 (0.841)
EQ-5D Index	-0.06 (0.730)	-0.07 (0.700)	0.06 (0.729)
EQ-5D VAS	0.06 (0.733)	0.23 (0.196)	-0.15 (0.414)

Notes - ^1^Spearman’s Rank Correlation Analysis, rho: Correlation Coefficient, #No statistically significant values, KOOS: Knee injury and Osteoarthritis Outcome Score, ADL: activities of daily living, Sport/Rec: Sport and Recreation Function, QoL: quality of life, IKDC: International Knee Documentation Score, SF-12: Short Form 12, MCS: mental component summary, PCS: physical component summary, EQ-5D: EuroQol-5D, VAS: visual analogue score

## Discussion

The main findings of this study are that ACLR is successful surgery when evaluating improvement of PROMs following the operation, but overall, graft tunnel placement does not have a significant impact on clinical outcomes.

Although previous studies, including Avadhani *et al*^9^ or Kazemi *et al*^19^, have shown that tunnel placement can influence patient outcome, the findings of this study contradict this theory. This study found that the positioning of the femoral tunnel according to the clockface method has no significant influence on patient outcome. This is not in line with the study conducted by Kazemi *et al*^19^, which showed that femoral tunnel positioning depending on the angle from the intercondylar notch influences outcome, specifically if at an angle between 30° and 40°. Nevertheless, the findings of this study concur with Gunaydin *et al*^20^ who did not find any significant differences in outcome for patients specifically with regards to EndoButton placement. Tibial tunnel placement was not found to have a significant influence on patient outcome, which is contrary to the majority of studies in this field including Avadhani *et al*^9^ and Padua *et al*^8^, which suggest that tibial tunnels placed too anteriorly along the tibial plateau result in worse outcomes. Nonetheless, the results of this study are not the first to contradict these findings and are in line with the conclusions of Behrend *et al*^6^. Graft tunnel angles were not found to be significantly correlated to post-operative PROM scores in this study, which is in line with the results found by Debnath *et al*^7^ suggesting no correlation for graft inclination.

Previous studies suggested that tibial tunnel placement in both anteroposterior (AP) and lateral views, graft inclination and femoral tunnel positioning in AP views were not found to have significant correlation with patient outcome^7^, which are in line with the results of the present study. A study by Kazemi *et al*^19^ suggests that patients with tunnels classified as low-posterior groups have better outcomes compared to low-anterior or high-posterior groups based on a combination of measurements in both AP and lateral views. EndoButton positioning on lateral radiographs does not significantly affect patient outcome according to the results of this study, which is in line with a retrospective study conducted by Gunaydin *et al*^20^.

Femoral tunnel placement along the intercondylar line in lateral view radiographs were significantly correlated with Lysholm scores, KOOS Sport/Rec sub-scores and KOOS Overall scores. However, the Spearman’s Rank Correlation Coefficients themselves demonstrate that the correlations are relatively weak (0.36 to 0.37). This does not confidently show that there is an association between tunnel placement and patient outcome scores one year following ACLR. These results are contrary to previous studies, including Behrend *et al*^6^ and Debnath *et al*^7^, finding that femoral tunnel placement in lateral view radiographs very significantly impact on patient outcome. Not only did the present study contradict findings suggesting posterior placement improves outcomes, it also contradicts the studies conducted by Biswal *et al*^4^ and Fernandes *et al*^5^, which suggest that posterior placement of the femoral tunnel improved patient outcomes.

Overall, this study did not find significant correlations between graft tunnel placement and post-operative PROM scores thereby suggesting that there may be other factors influencing patient outcome more profoundly than tunnel placement or that tunnel placement itself does not influence outcomes to the extent that previous studies might have found.

The strengths of the study include the large and comprehensive range of radiological measurements as this allows for a more holistic view of graft tunnel placement on both the AP and lateral anatomical planes and thereby gives a greater understanding of the impact of this on patient outcomes. It also included a wide range of validated PROM scores to evaluate clinical outcome. Moreover, the statistical testing of the pre-operative PROMs allowed for more confident interpretation of the post-operative scores. Including the pre-operative scores shows whether any significant differences were present prior to the operation and enables more accurate conclusions to be drawn. A limitation of this study was that all operations were performed by one specialist knee surgeon, thereby reducing the possible variation between graft tunnel placements. This does, however, remove any variability that might arise from different intra-operative technique, or approaches used by different orthopaedic surgeons. Another potential limitation is the relatively short follow-up time of one year. Many patients return to sport only after this time (as was the case in this study), which in-turn might influence the functional scores. Nonetheless, a baseline level of subjective function prior to return to sports is still important as it reflects generic activities of daily living within the first 12 months following surgery. Consideration of the limitations associated with using plain radiographs images to assess graft tunnel placement, both positioning and angles. Specifically, rotation of images might not be exactly replicated for each radiograph which may influence measurements and that only one individual assessed all the angles in this study. This could be improved upon by future studies by having a second independent assessor measuring all the positions and angles too. A potential limitation might be the inclusion of patients with both BPTB and hamstring grafts as well as patients with concomitant MCL or meniscal tears as these may alter or influence the patient’s subjective outcomes as well. However, this is a pragmatic clinical study which reflects ACLR and the common associated variables that accompany this in real-time practice. Another important factor to consider is that the surgical technique of anatomic ACL reconstruction involves both the tibial and femoral guide being aimed at the centre of the native ACL footprint. Due to an inherent degree of natural anatomic variation, this in itself could potentially influence graft tunnel positioning.

Future research using a larger cohort of patients with a multi-centre study, including objective physical examination findings with a longer post-operative follow-up timeframe would further build on the findings of the current study.

## Conclusion

ACLR is successful surgery when considering patients’ short-term self-reported functional outcomes. Graft tunnel placement does not significantly impact short-term clinical outcomes within the acceptable limits of technical variation in the procedure, acknowledging any degrees of freedom that may be permissible while still achieving optimal outcomes. This indicates that the surgical technique used for patients in this study allows for reliable tunnel positioning that positively influenced post-operative patient outcomes one-year following their surgery.
